# Exploring Adolescents' Understanding, Experiences and Beliefs About Pain: A Qualitative Study

**DOI:** 10.1111/hex.70132

**Published:** 2024-12-23

**Authors:** Isabelle Bogard, Julie Ayre, Jenna Smith, Joshua W. Pate, Andrew Sortwell, Jonah Gorringe, Georgia Gordon, Steven J. Kamper, Tie P. Yamato

**Affiliations:** ^1^ Faculty of Medicine and Health, School of Health Sciences The University of Sydney Sydney Australia; ^2^ Nepean Blue Mountains Local Health District Penrith New South Wales Australia; ^3^ Sydney Health Literacy Lab, Faculty of Medicine and Health, School of Public Health The University of Sydney Sydney Australia; ^4^ Graduate School of Health University of Technology Sydney Sydney Australia; ^5^ School of Health Science and Physiotherapy University of Notre Dame Australia Fremantle Australia; ^6^ Research Centre in Sports, Health and Human Development University of Beira Interior Covilhã Portugal; ^7^ Masters and Doctoral Program in Physiotherapy University of São Paulo City São Paulo São Paulo Brazil

**Keywords:** adolescents, chronic pain, health education, pain, pain education

## Abstract

**Background:**

Pain is prevalent across the lifespan and contributes to significant societal and economic burdens. The public often holds misconceptions about pain and pain management. Despite this, there are no well‐resourced public health initiatives delivering information about pain and pain management to the public. Adolescence is an opportune time to educate the public about pain. Health interventions designed for adolescents should reflect their understanding, beliefs and experiences; however, no studies explore this in non‐clinical populations of adolescents. We aimed to explore adolescents' understanding, experiences and beliefs about pain to inform the development of a school‐based pain education module.

**Methods:**

We conducted semi‐structured interviews with 25 adolescents in grades 7–10 (ages 11–16) attending Australian secondary schools. Interviews were conducted on video‐conferencing software, audio‐recorded, transcribed verbatim and analysed using Framework Analysis.

**Results:**

We generated three themes: (i) physical and psychological pain are distinct, (ii) psychological and contextual factors influence how someone feels or reacts to physical pain and (iii) physical pain matters if it impacts participation in meaningful activities.

**Conclusions:**

Adolescents' understanding and beliefs about pain do not always align with current scientific understanding of pain. School‐based pain education programmes should target these areas of misalignment. Addressing adolescents' misconceptions about pain through pain education could also create a more supportive school environment for adolescents experiencing pain. Interactive approaches to learning, such as discussions that encourage adolescents to reflect on their experiences of pain, could be a promising avenue for pain education.

**Patient or Public Contribution:**

Two co‐authors are part of the study population and contributed to the study design and analysis. Their input ensured the interview guide was appropriate for the target population and provided an adolescent perspective on the findings. They were remunerated for their time in accordance with consumer involvement guidelines.

## 1 Introduction

Almost everybody will experience pain at some point in life, and it is the primary reason people access medical care [1]. Pain is a subjective experience that influences and is influenced by biological, psychological and social factors to varying degrees [2]. For adolescents, pain can be associated with reduced physical activity [3], obesity [4], poorer mental health and increased substance use [5, 6] and impacts school attendance and performance [7, 8]. Chronic pain (pain that persists for three months or longer) is a significant global health issue in adults and children [9] and presents a substantial economic burden [10]. Misconceptions about pain are widespread on social media [11], among the general public [12] and among health professionals [13]. Despite the prevalence, societal and economic burden and widespread misinformation, no well‐resourced public education initiatives exist.

Adolescence, being a time of rapid social and neurological development, is an opportune time to educate young people about pain and how to manage it [14]. One in five children and adolescents experience chronic pain [15], with the prevalence of pain approaching adult levels by the end of adolescence [16]. Experiencing pain during adolescence is associated with experiencing chronic pain in adulthood [17], so intervening during adolescence is imperative. The National Strategic Action Plan for Pain Management identified the ‘development of an education program and resources for schools’ as a goal for pain management in Australia [18], however, none have been endorsed in Australian schools. For interventions targeting young people to be effective, they must be specifically designed and relevant for young people [14]. Hence, exploring adolescents' understanding, beliefs and experiences of pain is important for designing a successful pain education programme for this population group.

To date, research on adolescents' beliefs and understanding about pain has mostly been conducted in children with chronic pain and measures of beliefs have been developed in the same groups [19, 20, 21]. Further studies refer specifically to beliefs based on experience of a chronic pain condition [22, 23, 24]. Those instruments developed to measure pain understanding in general populations lack strong evidence to support reliability and validity [25, 26, 27]. Similarly, qualitative research aimed at understanding adolescents' perspectives on pain has predominantly been conducted in clinical populations. A qualitative study found children aged 8–12 with and without chronic pain had an injury‐focused perception of pain and minimised the role of emotional influences [28]. Another qualitative study of young adults aged 18–24 years with childhood‐onset chronic pain described the pain as indicating ‘something is wrong’ within the body, and persistent pain indicates an injury has not healed [29].

Research on experiences, beliefs and understanding of pain in adolescents without chronic pain is limited. A qualitative study found schoolchildren aged 10–16 predominantly attributed their pain to injuries and pathologies, followed by ergonomic issues and psychological factors [30]. This injury‐focused perspective is consistent with other qualitative research about beliefs and understanding of pain in clinical populations [28, 29], however further research is needed to better understand this research gap.

Our study aims to explore adolescents' understanding, experiences and beliefs about pain. These findings may inform the development of a school‐based pain education module for adolescents.

## 2 Methods

### 2.1 Study Design

We used a qualitative design using semi‐structured interviews with adolescents to explore their understanding, experiences and beliefs relating to pain. All study methods were approved by the University of Sydney Human Research Ethics Committee (protocol number 2023_403). The study protocol was registered on the Open Science Framework [31].

### 2.2 Participants and Recruitment

Eligible participants were students in grades 7–10 (ages 11–16) at Australian schools, chosen as this age group will receive the pain education module in future studies. The sample size of 25 participants was guided by information power [32], which estimates sample size considering aim, sample specificity, theoretical background, quality of dialogue and strategy for analysis [33]. We recruited a convenience sample using purposive and snowball sampling. Team members identified eligible participants from their personal contacts and extended social networks. These contacts were encouraged to share information about the study with other potentially eligible adolescents or their parents. We recruited an approximately even distribution of gender and school years. The team member or contact obtained verbal consent from the potential participant's parent to provide their contact details to the primary researcher (I.B.). We emailed the eligible participant's parent outlining the study and including the parent and adolescent participant information sheet. One follow‐up email was sent if the parent did not respond after one month. If the eligible participant and their parent agreed to participate in the study, an interview was scheduled at a time convenient for the participant. Participants chose if they wanted a parent present during the interview. Written informed consent was obtained from the parent and adolescent before conducting the interview.

### 2.3 Data Collection

We developed an interview guide to explore adolescents' experiences of pain and their understanding of pain in four key areas, (1) how pain is produced (i.e., biological processes associated with pain), (2) how internal and external factors affect pain, (3) the impact of pain and (4) management of pain (Supporting Information S1: File 1). The interview guide was developed by clinicians and researchers with expertise in paediatric pain (I.B., J.W.P., T.P.Y., S.J.K.) and qualitative research (J.A., J.S.). Two of the study authors (J.G., G.G.) were part of the study population and provided input on the interview guide. The guide included open‐ended, neutral, sensitive and understandable questions [34] and followed a line of questioning that encouraged participants to reflect and elaborate on their responses.

Interviews were conducted on video conferencing software, two interviews were changed to phone interviews due to software connectivity issues. Interviews were conducted between August 2023 and January 2024. The interviews were audio recorded and transcribed verbatim. I.B., a female PhD candidate and practising physiotherapist, conducted and transcribed all interviews. The transcripts were deidentified before analysis.

Sociodemographic information was collected verbally at the time of the interview and included; age, gender, Aboriginal or Torres Strait Islander Status, postcode (for socioeconomic status using Australian Socio‐Economic Indexes for Areas [SEIFA] [35] and Australian Statistical Geographical Classification [ASGC]), grade at school, school sector type, previous history of pain for three months or longer and previous education about pain (*operationalized as* ‘have you ever learnt about pain?’).

### 2.4 Data Analysis

Data were analysed using a Framework Analysis approach [36]. Consistent with a critical realist epistemology, we did not aim to determine whether participants had a ‘correct’ understanding of pain during the interview [37]. Rather, we aimed to describe and interpret a range of adolescents' experiences, understanding and beliefs about pain within their social contexts to inform the development of a school‐based pain education module. An inductive and iterative approach was used in the thematic analysis to maintain flexibility and generate rich and nuanced findings [38]. The team met regularly to enhance reflexivity and develop a deeper understanding of the data [39]. I.B. conducted and transcribed all interviews to ensure familiarity with the data. Using Microsoft Word, I.B. used open coding for the first 10 transcripts, then met with J.A. and J.S. who independently coded one transcript each. I.B., J.A. and J.S. compared coding labels and discussed and developed preliminary themes. After 19 transcripts were coded, I.B. met with the wider research team (J.A., J.S., J.W.P., A.S., T.P.Y., S.J.K.) to discuss the preliminary themes and then restructure and refine them based on feedback. After data collection was complete and preliminary themes were generated, the data were summarised in a Framework Matrix. The matrix was developed on Microsoft Excel, where rows were participants, columns were aspects of themes and cells were summarised data (quotes) [38], with one theme per sheet. Using this systematic approach, we were able to recognise patterns in the data, including identifying any contradictory data and deviant cases [38]. The framework also ensured sufficient evidence for each theme and facilitated further refinement. I.B. met with J.G. and G.G. to discuss the interpretation of themes from an adolescent perspective. Data analysis was finalised from the writing process itself.

## 3 Results

Twenty‐five participants were interviewed: 19 were identified by personal contacts of the researchers and six by other study participants. Four participants chose to have one parent present during the interview, with parental input varying from no input to actively participating in the interview. Parental perspectives were not included in the data analysis. The interviews lasted between 16 and 39 min (mean = 26 min).

### 3.1 Sociodemographic Characteristics

The average age of the sample was 13.7 years (SD 1.2), and 13 of 25 participants were female. No participants identified as Aboriginal and/or Torres Strait Islander. There was a roughly even distribution of participants in each age group and 12 attended independent schools. Almost all (24/25) participants lived in major cities and high socioeconomic areas (median = 10, IQR = 0.5). Most (18/25) participants reported they had received education about pain previously, and nine reported they had previously experienced pain for three months or longer (Table 1).

**Table 1:** Participant characteristics.

Variable	*n* = 25
Age (years), mean	13.7
Gender (female), *n*	13
Socioeconomic status, SEIFA index/10^a^, median	10
Geographic area, ASGS^b^, *n*	
Major cities	24
Inner regional	1
State or territory in Australia	
New South Wales (NSW)	24
Queensland (QLD)	1
School grade, *n*	
Grade 7	6
Grade 8	6
Grade 9	7
Grade 10	6
School sector type, *n*	
Government	8
Independent	12
Catholic	5
Experienced pain three months or longer—yes, *n*	9
Previous education about pain—yes, *n*	18

^a^ Socioeconomic status was calculated on the SEIFA index (1 = lowest socioeconomic decile, 10 = highest socioeconomic decile).

^b^ Based on the Australian Statistical Geographical Classification (ASGC), 2016.

### 3.2 Themes

The interviews generated three themes: (i) physical and psychological pain are distinct, (ii) psychological and contextual factors influence how someone feels or reacts to physical pain, and (iii) physical pain matters if it impacts participation in meaningful activities (i.e., activities they perceive to be important). The focus on physical pain in themes two and three is because participants mostly discussed experiences of physical pain, and the pain education module will relate to what they conceptualise as physical pain.

#### 3.2.1 Theme 1: Physical and Psychological Pain Are Distinct

Participants discussed physical and psychological pain as distinct entities. Despite describing a range of experiences, participants predominantly described physical pain in terms of injury. This was associated with beliefs that physical pain is visible to others, localised to a part of the body and heals within a short period of time. By contrast, psychological pain was hidden from others, felt throughout the body and harder to manage. These beliefs also influenced their care‐seeking behaviours and management strategies.

When asked about their experiences of pain, most participants described injuries, such as grazed skin and broken bones. Fewer participants described pain due to excessive sport, ‘growing pains’, ‘period pain’ and abdominal pain. Despite a range of physical pain experiences, physical pain was analogous to injury for most participants:

It feels simple, you get hurt, you feel pain. It's there to know that you've become hurt. [P18, 14M]

Participants described mental and emotional (‘psychological’) pain as being related to grief, negative emotions, stress and depression. Psychological pain was conceptualised as predominantly emotional and cognitive, whereas physical pain was conceptualised as predominantly sensory:

Physical pain you can actually feel it, but emotional pain, you don't feel, you just think it. [P13, 12M]

Participants suggested physical pain was more localised to one part of the body, whereas psychological pain was more pervasive throughout the body:

I feel like physical pain… you're only feeling it in one part of your body, but I feel like with emotional pain, you're feeling it through your whole body. [P9, 14F]

Some participants suggested physical pain was easier to understand because it is visible to others, whereas psychological pain was described as an internal experience, meaning others can only understand it if the person chooses to ‘open up’:

Physical pain, it's more on like the outside of the person, so it's more opened up to … like you could physically see any injuries … mental pain is inside, and no one can see anything and, the only way people can like realise is if the person experiencing the mental pain opens up. [P21, 15M]

Consistent with this belief, many participants suggested psychological pain was harder to manage and more persistent than physical pain:

Emotional pain… you can't do anything to just like fix it … you're hurt more mentally, and that's much harder to like … make feel better. [P14, 14F]

By contrast, participants expected physical pain to be temporary and fixable:

Physical pain, it's going to be fixed…it doesn't last a long time, it kind of lasts like short periods and…you're only feeling it in one part of your body. [P9, 14F]

As such, many participants described ignoring initial experiences of physical pain. They often reported delaying seeking help until physical signs of injury developed or worsened (e.g. swelling, bruising), if the pain persisted longer than expected, or if the pain restricted them from daily activities or hobbies. Persistent pain indicated an injury was serious, had not healed properly, or required further investigations. However, they suggested psychological pain is likely to get worse, so should be addressed sooner:

With physical pain, if you don't address it initially in some cases it's fine, and it heals by itself, but obviously if it's something bad and you don't address it initially, it just gets worse and worse, like you overuse yourself for something. And then with mental pain, I think it will just always get worse. So, I think to address it initially is what you need to do. [P4, 14F]

Most participants sought help from doctors and physiotherapists to manage their physical pain. They predominantly used simple management strategies such as RICE (rest, ice, compression, elevation), first aid, simple analgesics (paracetamol, ibuprofen) and distraction. Very few used psychosocial strategies, such as talking to others or relaxation techniques, to manage their physical pain. However, participants described how psychosocial strategies were important for treating psychological pain:

Physical pain would be like medication, like looking after the injured thing, it's more like just treating it, whereas mental pain, it's like treating it within … talking about it can help … just kind of like, acknowledging the pain's there. [P23, 15M]

Despite most participants contrasting physical and psychological pain, some participants acknowledged that physical pain and psychological pain can interact. They suggested that some painful experiences such as headaches and bullying could be both physical and psychological in nature:

Bullying's kind of verging on both mental and physical, because if you're getting hit, that's physical pain, but also the mental side effects of that. [P24, 15M]

While participants had distinct beliefs and understanding about the mechanisms, experience and management of physical and psychological pain; some suggested the overall purpose of experiencing physical and psychological pain was similar:

If you feel like mentally pain, like, let's say you're really sad or something, your body telling itself that something's not right or like, physical pain when you get cut on your leg, something's not right. [P24, 15M]

Physical pain was predominantly discussed as separate to psychological pain. Despite this separation, the following two themes explore how participants perceived psychological and contextual factors to influence and be influenced by physical pain.

#### 3.2.2 Theme 2: Psychological and Contextual Factors Influence How Someone Feels or Reacts to Physical Pain

Participants described how psychological and contextual factors can influence how someone feels and reacts to physical pain. Two contrasting beliefs underpinned this; that people feel physical pain differently, or people feel physical pain in the same way but react differently.

Participants described how psychological factors such as emotions and thoughts could influence how pain feels. Many participants described various ways that emotions and thoughts could amplify or decrease the intensity of their pain. This included emotions that were directly related to the circumstances of the injury:

If you have a really bad injury … this sadness comes up and you, as well as the pain, and you're kind of getting hit by both at the same time, you could feel like you're feeling more pain. [P18, 14M]

As well as someone's general emotional state:

Cause if you're happy, and getting cheered up, you don't really feel pain … but if you're really sad and down, I think you're much more susceptible to pain and you're much more affected by it. [P22, 16M]

Participants described how thinking about pain, being ‘paranoid’ about hurting yourself more [P22, 16M], or worrying that the pain may not get better, could make pain feel worse:

I reckon thoughts can increase the receptors, like your vulnerability, to experience the pain or not. [P24, 15M]

Participants also described how contextual factors, such as social support, could influence how much pain someone feels:

Who you're surrounded with, your friends and family, how they support you with it, that could impact you mentally and then internally impacts how much pain you feel. [P22, 16M]

Previous experience of pain was another contextual factor, however there were varying beliefs about how this influences the feeling of pain. Some participants suggested that repeated experiences of pain could make pain feel worse, whereas others suggested experiencing persistent pain would make someone less sensitive to it:

He [my friend] has known his pain, like pretty much his whole life, so he's probably almost getting used to it now, and so it probably doesn't feel as bad to him. [P20, 13M]

In contrast to the belief that people feel pain differently, other participants suggested the same injury would result in the same amount of pain for everyone, but people may react differently to the pain:

You can react different, but I think you feel the same thing. [P13, 12M]

Reactions to pain could also be influenced by psychological and contextual factors. Participants described how psychological attributes such as mindset, thoughts and coping could influence how someone reacts to pain:

The resilience of the person, maybe like the coping mechanisms the person has, and the general mindset of the person. [P23, 15M]

The ability to tolerate pain was associated with positive psychological attributes such as coping, resilience and a ‘pain‐enduring headspace’ [P24, 14M]. Conversely, someone who has a more visible reaction such as crying may be perceived as ‘over‐reacting’ (P4, 14F) or ‘weak’:

People might call you a bit of a cry‐baby or something like that…I guess this is like a sign of weakness a bit. [P23, 15M]

Participants also discussed how contextual factors such as family, societal expectations, or pressures to return to sport could influence how someone reacts to pain:

If you grow up around like a lot of like siblings and you're always getting knocked about, you kind of learn to get over it [pain]. Whereas if you don't, I feel like it's a lot harder for you to grow that painful tolerance. [P4, 14F]

To a lesser extent, some participants described how physical attributes such as skin thickness, nerve conditions, age and genetics could influence the experience of pain:

Say someone got a splinter… I don't think it would hurt as much for people with thicker skin than thinner skin. [P16, 13F]

Overall, participants in the study discussed how psychological and contextual factors could affect the experience of physical pain, however, there were varying beliefs as to whether pain felt different for each person, or whether people just reacted differently.

#### 3.2.3 Theme 3: Physical Pain Matters If It Impacts Participation in Meaningful Activities

Participants were often unperturbed by pain, irrespective of the severity, cause, or persistence. However, pain seemed to matter if it prevented them from doing what they wanted to do in the present or threatened their ability to do so in the future.

Participants described frequent experiences of pain that were often the result of minor injuries, such as rolling an ankle, a collision in sport or grazed skin. Participants who experienced these frequent and minor injuries were often unperturbed by recurrent experiences of pain, as the impacts of the pain were often temporary and inconsequential:

About two months later… I felt the pain again… I was like, ‘oh, why did it happen again?’ And then I thought about like the process of the pain and I thought, ‘oh, it's not too bad and I get to miss school for a bit’. [P7, 12M]

Even with seemingly substantial injuries, such as broken bones, participants often described little impact or concern:

I was lucky because it was just my wrist so the cast only went up to around here [gestured to forearm] and we got a waterproof one and it had Velcro so I could take it on and off. So, I could horse ride, and zip‐line, and swim. So, I wasn't impacted much at all. [P4, 14F]

Their attitude toward the pain did not seem to correlate with the ‘severity’ of the injury, rather how much it interfered with their ability to engage with meaningful activities:

It's like how I injured my left hand, it hurt because I couldn't do what I wanted to be able to do. [P18, 14M]

Notably, injuries that impacted sports participation or performance were the most upsetting for participants in this study:

I was devastated. I was captain of the team, we were playing one of the worst teams in the league and it was just sad and was … just annoying really, it wasn't really a good experience and didn't really help with my confidence. [P10, 12M].

Participants worried about pain if it was new or unexpected, more severe than usual or if it persisted longer than expected. The worry seemed to be underpinned by the belief that this pain could threaten their ability to do the things they enjoy in the future:

I like to play netball a lot so I don't want it to impact playing netball and I don't want it to get super serious so I can't play sport again. [P25, 12F]

This attitude toward pain was shared by participants who experienced persistent pain. Experiences of persistent pain included frequent headaches, chronic widespread musculoskeletal pain, recurrent menstrual pain and specific conditions such as Osgood‐Schlatter's disease. They described the constancy of pain as ‘annoying’, but it often had limited impact on daily functioning and physical activity:

It's just annoying. I'd prefer it wasn't there, but it doesn't change what I do. It's a constant thing, so it can't impact me because it's always there. [P5, 14F]

These participants also described the pain as upsetting or concerning when it stopped them from doing what they enjoyed:

When it's really bad it irritates me more… I have to sit out on a sport sometimes, like when it was really bad at dance. [P3, 15F]

While both participants with acute injuries and chronic pain described their pain as having minimal impact on physical functioning, there were distinct differences in how the pain impacted them socially. Many participants reported the positive impact of feeling supported by family and friends after they had injured themselves, such as receiving messages of support and being physically assisted at home and school. However, some participants with chronic pain reported the negative impact of not feeling understood and judged by their peers:

They were always just questioning why I was sitting out of class, or why my eyes would go watery sometimes. And they just didn't know how much pain I was in really, or the cause of it, or why I was sitting out. They thought I was just trying to bludge class. [P3, 15F]

For the participants in this study, it seemed that pain mattered because of its impact on meaningful activities. Acute and chronic pain mattered if it stopped them from doing what they wanted to do, or if it threatened their ability to do it in the future. However, there were differences in how participants with acute and chronic pain discussed the social impact of pain.

## 4 Discussion

Participants in this study consistently distinguished between psychological and physical pain, described differences in the experience of physical pain due to psychological and contextual factors, and felt pain mattered if it impacted their participation in meaningful activities. These findings highlight areas where adolescents' understanding of pain do not align with the current scientific understanding of pain and identify key areas to address with school‐based pain education.

Participants predominantly associated pain with injury and described physical pain in terms of injury. This finding is consistent with other qualitative studies of young people with and without persistent pain [28, 29, 30]. Injuries were the most common pain experience for our participants and are prevalent among young people [40]. They are the leading cause of emergency department visits and the second most common reason for hospital admissions among children and adolescents [40]. As such, an injury‐focused understanding of pain is unsurprising, given that individuals learn their concept of pain through life experiences [2], and children often communicate their understanding of pain based on their own experiences [28]. Misconceptions about pain and injury, as summarised in Table 2, may also be reflective of societal misconceptions about pain [12, 13].

**Table 2:** Comparison of study findings and scientific understanding of pain.

Study finding	Scientific understanding of pain	Aligned or misaligned
Physical and psychological pain are distinct.	Pain is a sensory and emotional experience [2].	Misaligned
Physical pain is visible to others, localised to one part of the body, and is short‐term.	Pain and nociception are not the same. Pain is a subjective experience, and nociception is a process in the central nervous system [2]. Neither are visible; however, signs of an injury may be visible.	Misaligned
Physical pain is mostly due to injury.	Pain may or may not be associated with tissue damage (injury) [2].	Misaligned
Pain that persists beyond the expected time frame for recovery indicates serious injury, delayed healing or requiring further investigation.	Injury occurs when there is damage to body tissues, which may or may not be associated with pain [2]. Body tissues typically heal within a defined time frame, but pain can persist beyond this time frame. This persistent pain can be due to ongoing disease processes or changes in the peripheral and/or central nervous systems [41].	Misaligned
Psychological and contextual factors can influence how one feels and reacts to pain.	Pain is understood through the biopsychosocial model [41].	Aligned
Physical pain is predominantly managed using biomedical strategies (e.g., RICE, medications)	Psychological and social strategies are also useful in managing physical pain [9].	Misaligned
Some people can ‘tolerate pain’ better than others. People who can tolerate pain are more resilient.	Pain is a subjective, individual experience that is influenced by physical, psychological and social factors [2, 41].	Misaligned

In this study, participants reported not worrying about pain unless it impacted their ability to participate in meaningful activities; the inability to engage in sports and play was particularly concerning for participants. However, the minor worry reported by our participants, regardless of whether they reported pain, is in stark contrast to findings of research from chronic pain populations. A qualitative study of children attending a chronic pain clinic reported significant worry and distress associated with the unpredictability of their pain, uncertainty about the underlying cause, and repeated treatment attempts [42]. Experiences of worry about pain may differ between clinical and non‐clinical populations of children, hence caution should be used when interpreting our study findings with reference to populations from clinical contexts.

School‐based pain education should target discrepancies in adolescents' understanding of pain and contemporary scientific understanding of pain. These discrepancies are summarised in Table 2. Pain education should broaden adolescents' understanding of pain beyond injuries. An injury‐focused understanding of pain is inadequate for explaining persistent or recurrent pain, which could lead to seeking non‐evidence based investigations and treatment for pain [43]. Pain education could also explore how psychosocial strategies can be useful for managing pain. Participants discussed how pain mattered if it impacted their ability to participate in meaningful activities, but mostly relied on biomedical management strategies. Learning new information could expand their options for managing future experiences of pain and reduce its impact on meaningful activities.

Learning about pain at school could reduce the stigma associated with chronic pain. Adolescents with chronic pain, including participants in our study, report feeling stigmatised by peers and school personnel [44] due to perceptions that they are ‘faking or exaggerating their pain for attention’ [45]. Some participants in our study perceived the ability to ‘endure’ pain as being resilient, whereas reacting to pain was seen as ‘weak’. Pain education should address the misconceptions about pain that may underpin stigma, such as pain is short‐term and visible. Developing a greater understanding of chronic pain could create a more supportive school environment, which is important for adolescents experiencing chronic pain [44].

Facilitating discussions about adolescents' experiences of pain could be a promising avenue for developing a biopsychosocial conceptualisation. The interview guide and follow‐up probes encouraged participants to reflect and elaborate on physical, psychological and social factors that influenced their experience of pain. Indeed, some participants in our study reflected that they had not discussed pain ‘in this way’ before the interview. Interactions between people can often enhance an individual's learning [46]. While we cannot suggest the interviews facilitated learning or a conceptual shift for the participants, it did facilitate a broader discussion about pain that is more consistent with a biopsychosocial understanding. The Interactive‐Constructive‐Active‐Passive (ICAP) framework is a constructivism‐based learning framework [47], and has been applied in other pain education programmes [48]. The framework suggests interactive activities (e.g., discussions) lead to greater engagement, deeper understanding and greater retention of information compared to other learning activities [47]. Therefore, using pedagogical approaches that encourage student interaction in the learning process, such as class discussions and partner work, could be an effective approach for the pain education module. This approach could lead to greater engagement with the content and possibly lead to a greater understanding of how biopsychosocial factors influence pain.

Having a diverse research team was a strength of this study. The authors included researchers with expertise in various areas, including paediatric pain, qualitative methods and school education, clinicians with experience managing adolescents with pain, and adolescents who are part of the study population. This ensured the study methods were appropriate for the population and generated rich and nuanced findings. Further, having three researchers familiar with and coding the transcripts facilitated a deeper understanding of the data. The sampling method also ensured an equal distribution of gender and school year.

The recruitment strategy means our sample may not reflect the broader Australian secondary school student population to whom the pain education will be delivered. We recruited participants from our social networks, who were more likely to be in higher socioeconomic deciles, live in metropolitan areas and have greater access to education, than the general Australian adolescent population [49, 50]. As such, our results may not accurately reflect the experiences and understanding of pain for students who live in regional or remote areas of Australia, live in lower socioeconomic deciles, or identify as Aboriginal and Torres Strait Islander. These perspectives are important, as social factors such as socioeconomic status are associated with chronic musculoskeletal pain in childhood and adolescence [51]. The lack of information about cultural diversity is also an important limitation in this study, as the findings may not capture the pain experiences and beliefs of adolescents from different cultural backgrounds. Additionally, some of the study findings may be an artifact of the interview. The interview encouraged participants to reflect on factors that influenced their experience of pain, which we could have interpreted as a greater biopsychosocial understanding of pain. The focus on injuries could have been because the participants were aware the interviewer was a physiotherapist.

Future research should consider the experiences, beliefs and understanding of pain in adolescents with diverse cultural backgrounds and contexts to ensure that pain education is relevant to all students to whom it will be delivered. While school‐based pain education is an important starting point for addressing the stigma associated with chronic pain, a more comprehensive approach including a diverse range of families, teachers and sports coaches is needed.

## 5 Conclusions

School‐based interventions could play a role in developing adolescents' understanding of pain. Education programmes should target areas where adolescents' understanding of pain does not align with current scientific knowledge. Encouraging adolescents to reflect on and discuss their experiences of pain could possibly lead to a greater understanding of how biopsychosocial factors influence pain. By addressing misconceptions and promoting psychosocial pain management strategies, pain education could also address the stigma associated with chronic pain, ultimately fostering a more supportive environment for those affected.

## Author Contributions

**Isabelle Bogard:** conceptualisation, investigation, writing–original draft, methodology, formal analysis, writing–review and editing. **Julie Ayre:** methodology, writing–review and editing, formal analysis. **Jenna Smith:** methodology, writing–review and editing, formal analysis. **Joshua W. Pate:** writing–review and editing, methodology, formal analysis. **Andrew Sortwell:** methodology, writing–review and editing, formal analysis. **Jonah Gorringe:** methodology, formal analysis. **Georgia Gordon:** methodology, formal analysis. **Steven J. Kamper:** conceptualisation, investigation, funding acquisition, writing–review and editing, methodology, formal analysis, supervision. **Tie P. Yamato:** conceptualisation, investigation, methodology, writing–review and editing, formal analysis, supervision.

## Ethics Statement

Ethics approval for this research was obtained from the University of Sydney Human Research Ethics Committee [Approval No. 2023_592], and informed consent was obtained from all participants.

## Conflicts of Interest

The authors declare no conflicts of interest.

## Supporting information

Supporting information.

## Data Availability

Data can be made available on request to corresponding author subject to requirements from the University of Sydney Human Research Ethics Committee.
