# Supplementary material for: Exploring Adolescents' Understanding, Experiences and Beliefs About Pain: A Qualitative Study
Source: Health Expect. 2024 Dec 23;27(6):e70132. doi: 10.1111/hex.70132 (PMC11666834; doi:10.1111/hex.70132)
Supplement: Supplementary file 1 — Supporting information. [file HEX-27-e70132-s001.docx]

| **Question** | **Alignment with key areas** |
| --- | --- |
| Tell me about a time you experienced pain | *Initiate dialogue* |
| How did you react? Why?  How did other people (family, friends) react? Why? | 4) Management of pain  3) How internal and external factors affect pain |
| What do you think was happening in your body (when you were experiencing pain)?  What do you think the pain is trying to tell you? | 1) How pain is produced |
| What did you do when you felt the pain? Why?  How did that affect the pain? | 4) Management of pain  3) How internal and external factors affect pain |
| Did the pain affect other things in your life? In what way? | 3) Impact of pain |
| Did you notice some things made the pain worse or better?  What sort of things? | 3) How internal and external factors affect pain |

Supplementary File 1: Interview guide and alignment with key areas
